# *Hylotelephium spectabile*, a New Host for Carnation Tortrix Moth (*Cacoecimorpha pronubana*) and Molecular Characterization in Greece

**DOI:** 10.3390/insects12030245

**Published:** 2021-03-15

**Authors:** Konstantinos B. Simoglou, Dimitrios N. Avtzis, Joaquín Baixeras, Ioanna Sarigkoli, Emmanouil Roditakis

**Affiliations:** 1 Department of Quality and Phytosanitary Inspections, Rural Economy & Veterinary Directorate of Drama, 66133 Drama, Greece; sarigkolii@pamth.gov.gr; 2 Forest Research Institute, Hellenic Agricultural Organization Demeter, Vassilika, 57006 Thessaloniki, Greece; dimitrios.avtzis@fri.gr; 3 Institut Cavanilles de Biodiversitat i Biologia Evolutiva, Universitat de València, Carrer Catedràtic José, 46980 Paterna, Spain; joaquin.baixeras@uv.es; 4 Department of Agriculture, School of Agricultural Sciences, Hellenic Mediterranean University, 71410 Heraklion, Greece

**Keywords:** *Cacoecimorpha pronubana*, *Hylotelephium spectabile*, molecular characterization, new host

## Abstract

**Simple Summary:**

*Hylotelephium spectabile* (Saxifragales, Crassulaceae), a widespread ornamental plant was found infested by larvae of *Cacoecimorpha pronubana* (Lepidoptera, Tortricidae) a highly polyphagous pest of a wide range of crop, as well as ornamental plants. To the best of our knowledge, this finding suggests that *H. spectabile* should be considered a new host plant for *C. pronubana*. Moreover, molecular characterization of the Greek pest population place it along with European species clade.

**Abstract:**

*Cacoecimorpha pronubana* (Hübner) (Lepidoptera, Tortricidae) is a highly polyphagous pest of a wide range of crop and ornamental plants. It is of Mediterranean origin and widespread in European and Mediterranean Plant Protection Organization (EPPO) region. For the first time, infestations of *Hylotelephium spectabile* (Boreau) Ohba (syn.: *Sedum spectabile* Boreau) (Saxifragales, Crassulaceae) ornamental plants by *C. pronubana* larvae, in private gardens in urban area of Drama, Greece, were found. Species identification was conducted based on morphology of female genitalia. In addition, due to reports on occurrence of cryptic *C. pronubana* species within Europe, DNA barcoding was carried out to determine the molecular status of the pest. This communication reports a new host of *C. pronubana* and places the Greek pest population along with European species clade.

## 1. Introduction

The carnation tortrix moth, *Cacoecimorpha pronubana* (Hübner) (Lepidoptera, Tortricidae) is a highly polyphagous and locally important pest of a wide range of crop plants. In addition, *C. pronubana* infests also ornamental plants (Table 1). Usually considered a typical Mediterranean element, *C. pronubana* is widely distributed among most EPPO member-countries [1] and is locally common across Europe to Asia Minor, North Africa, but it is also recorded from South Africa as well as North America [2,3,4,5,6,7]. In Greece the species occurs both in mainland [7] and on the island of Crete [8]. 

*Hylotelephium spectabile* (Boreau) Ohba (syn.: *Sedum spectabile* Boreau) (Saxifragales, Crassulaceae) is an ornamental plant species that is widely distributed throughout the warm and temperate zones, originating from Korea and Northern China [13]. It is approximately 45 cm high, glaucous green in color and bears large, flat-topped, pink-purple inflorescences that attract nectar-feeding insects (particularly butterflies) hence it is commonly known as butterfly stonecrop. *Hylotelephium spectabile* has sparsely toothed leaf margins and flower stamens longer than petals, which differentiates it from other species in the genus [14].

In this study, we report a carnation tortrix infestation on butterfly stonecrop plants. Based on an extensive literature review, to the best of our knowledge no Crassulaceae member-plants have been previously reported as host of this species. Our results support that the particular plant species can be considered a new host of *C. pronubana* [6,15].

## 2. Identification

**Figure 1:**
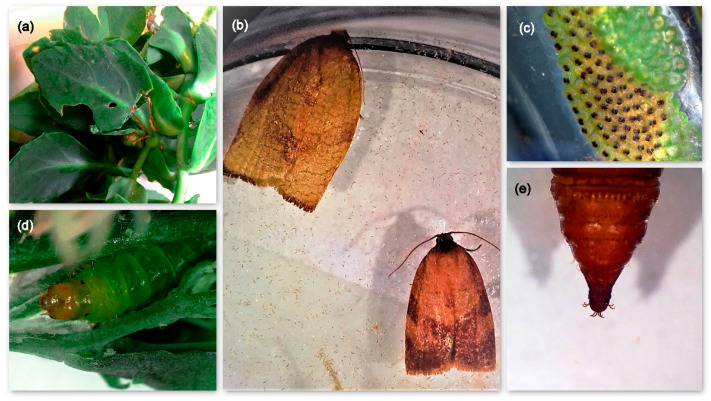
(**a**) Butterfly stonecrop plant leaves, chewed and knitted together by *Cacoecimorpha pronubana* larvae; (**b**) Female (left) and male (right) individuals of *Cacoecimorpha pronubana*; (**c**) Eggs mass of *Cacoecimorpha pronubana* laid on petri-dish; (**d**) *Cacoecimorpha pronubana* larva on butterfly stonecrop plant inflorescence; (**e**) *Cacoecimorpha pronubana* pupa cremaster with 8 hooked bristles.

In mid-August 2020, caterpillars of an unknown species have been detected feeding on potted butterfly stonecrop plants located in private gardens in the broader area of Drama (Region of Eastern Macedonia and Thrace, Northern Greece). Infestation had caused considerable damage on foliage as well as on blossoms, reducing the ornamental/aesthetical value of the butterfly stonecrop plants. Leaves of the infested plants were chewed and knitted together with silken webbing, in which feeding caterpillars inhabited (Figure 1a). This observation is in line with the first description of carnation tortrix by Fisher (1924) [16], who stated that caterpillars older than the 3rd instar feed under a dense silken cover, remaining completely hidden in the rolled leaf or leaves from which they are feeding.

**Figure 2:**
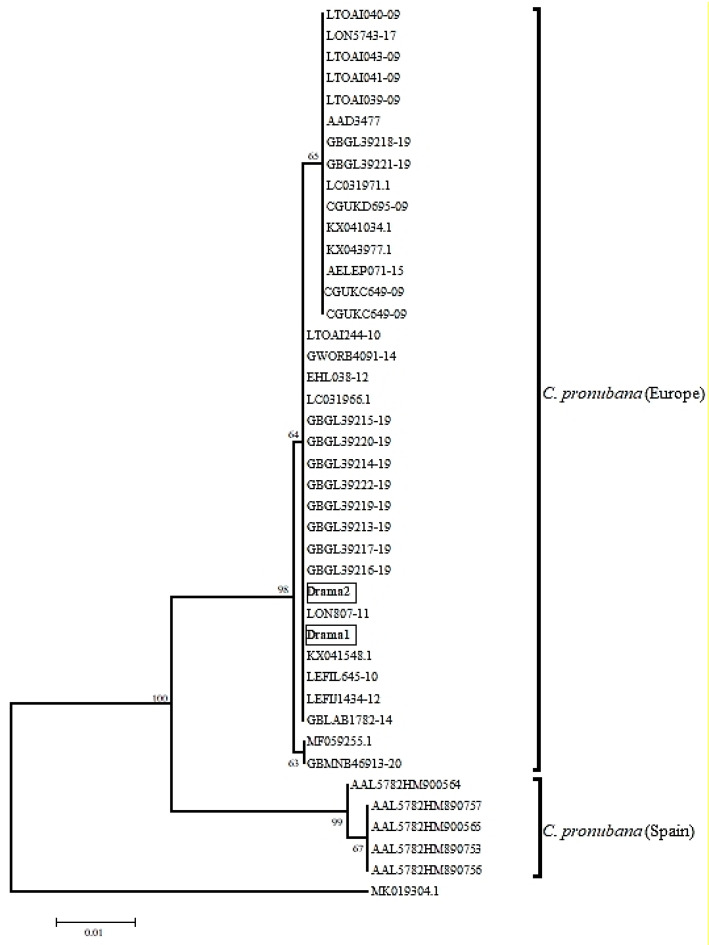
Rooted NJ phylogenetic tree (500 bootstrap replicates / K-2P) that contains the two *C. pronubana* sequences from Greece (Drama 1 and 2) and all the available *C. pronubana* sequences in NCBI and BOLD databases. Scale-bar stands for 0.01 K-2P distance.

Larvae collected in the field were reared in the laboratory of the Department of Quality and Phytosanitary Inspections of Drama (Drama, Greece). The external morphological characters of eggs, larvae, pupae and the emerged adults resembled those of *C. pronubana* [3,11,16,17] (Figure 1b–e). Morphological identification based on female genitalia was conducted by one of the authors (J.B.) and compared with literature reports and collection samples. Further on, due to reports on occurrence of cryptic *C. pronubana* species within Europe [18], DNA was extracted from the specimens in order to proceed with DNA barcoding. To do that, two individuals (4th instar caterpillars) were stored in 95% ethanol and posted for analysis to the Laboratory of Forest Entomology (Forest Research Institute, Hellenic Agricultural Organization Demeter) (Thessaloniki, Greece). Total genomic DNA was extracted from each individual separately, using PureLink^®^ Genomic DNA kit (Invitrogen, Waltham, MA, USA) and following the manufacturers’ instructions modified during the tissue grinding process [19]. Polymerase Chain Reaction (PCR) was run in 25 μL volumes with primers LCO/HCO that amplify a 658bp-fragment of mtDNA’s Cytochrome Oxidase subunit I gene (COI) [20]. PCR procedure is provided in Avtzis et al., (2021) [19]. The purification of PCR products was performed with PureLink^®^ PCR Purification Kit (Invitrogen) following the manufacturer’s protocol and purified products were sequenced in the automated sequencer ABI3730XL of CeMIA Company (Larisa, Greece), using the same primers as in PCR. Finally, sequences were initially visualized with Chromas Lite^®^ version 2.01 (Technelysium Pty Ltd., South Brisbane, Australia) and after removing the ambiguous nucleotides at both ends of the sequences, a final product of 443 bp long was obtained for each individual. These sequences were then blasted in the NCBI GenBank database (https://www.ncbi.nlm.nih.gov/genbank/ (accessed on 13 March 2021)), placing them among the sequences of *C. pronubana* deposited there. In particular, both these sequences were 100% identical with *C. pronubana* from France (Accession Number: KX041548) and Italy (Accession Number LC031966), and 99.77% similar with the sequences from the United Kingdom (Accession Number: KX043977), Germany (Accession Number: KX041034) and Italy (Accession Number LC031971). Given the distinct separation that has been previously revealed among the populations of *C. pronubana* worldwide [18], we compiled all the available *C. pronubana* sequences, deposited both in NCBI (see Accession Numbers above) and BOLD (BOLD BINs: AAD3477 and AAL5782) into an integrated large dataset and constructed a Neighbor-Joining phylogenetic tree. To do that, we used MEGA6 [21], employing K-2P distances with 500 bootstrap replicates, and rooted the tree with a sequence of a closely related tortricid species (Accession Number MK019304.1). Greek sequences were apparently assigned to the clade that contains all European *C. pronubana* sequences, remaining distinctly separated from those of Spain (Figure 2). Interestingly, divergence between Spanish and Greek (and the other European) sequences is found to be much higher (>3.5%) than commonly expected for intraspecific divergence within tortricid species (e.g., *Anarsia lineatella / Grapholita molesta* [22]). Nevertheless, as deep intraspecific barcode divergences have already been detected in a few more tortricid species as well [23], this finding should be further investigated to conclude whether this pattern is an artifact of non-continuous distribution or by-product of mitochondrial evolution [24].

## 3. Biological and Taxonomic Notes of *Cacoecimorpha pronubana*

*Cacoecimorpha pronubana* adults are nocturnal and males are much more active than females with a flight that is erratic and agitated [16]. *Cacoecimorpha* Obraztsov 1954 is a monotypic tortricid genus with adult females having a wingspan of 18–22 mm, with pale orange brown forewings that are reticulated with darker brown coloration, while hindwings are mainly orange. Adult males are generally smaller (wingspan of 12–17 mm) and their forewings are orange brown with reddish-brown and purplish-black markings while their hindwings are bright orange with blackish border (Figure 1a). Both male and female genitalia give key diagnostic characters [25,26]. The eggs are light green and are laid in egg-masses [3] in which around 200 eggs are overlapping and surrounded by a mucilaginous substance [16] (Figure 1c). Caterpillars are olive to bright green with slightly paler pinacula and a length that can reach up to 20 mm. The head is greenish yellow or yellowish brown, with dark brown marks and the prothoracic and anal plates have green coloration, with dark brown marks (Figure 1d). The anal comb is green and is usually 6-pronged. Pupae are 9–12 mm long, with brownish black to black colors. They have elongated and tapered cremaster, with 8 strong, hooked bristles [3] (Figure 1e). In Europe, the carnation tortrix moth has two to five generations per year, with adults emerging from April to October, or even later. The eggs are laid on the leaves and hatch about 15–20 days later [3]. The duration of egg-laying of a single female is approximately 12–14 days during which 7 or 8 egg-masses are laid [16]. Caterpillars feed on leaves, inflorescence and fruits and go through seven instars. Pupation takes place in rolled leaves or amongst webbed foliage and lasts approximately 13–17 days. First instars usually overwinter sheltering on the foodplant inside silken hibernacula. However, under favorable conditions feeding may continue through winter and then all stages of the pest may be found together [3,16]. 

## 4. Conclusions

Hereby, a new host plant was detected for *C. pronubana*. Therefore, the pest list of butterfly stonecrop, *H. spectabile*, has to be updated, including the carnation tortrix moth. Our findings can significantly contribute to the accurate pest identification for ornamentals in future and of butterfly stonecrop, in particular. Since both the pest and its new host are wide spread, future infestations or outbreaks can occur anywhere in the world, thus current information will facilitate rapid and appropriate pest management actions in the future. In addition, DNA barcoding confirmed the findings of Gilligan et al. (2020) [18], placing Greek carnation tortrix moth population along with European species clade, establishing further the knowledge on *C. pronubana* in Europe.

## Figures and Tables

**Table 1:** Non extensive host-plant list of *Cacoecimorpha pronubana*.

Host Category	Common Name	Scientific Name	Botanic Family	References
Crop plants	strawberry	*Fragaria* × *ananassa* Duchesne	Rosaceae	[3,6,9]
	almond	*Prunus dulcis* (Mill.) D.A. Webb	Rosaceae	
	cherry	*Prunus avium* L.	Rosaceae	
	apple	*Malus domestica* Borkh.	Rosaceae	
	raspberry	*Rubus* spp.	Rosaceae	
	grapevine	*Vitis vinifera* L.	Vitaceae	
	citrus	*Citrus* spp.	Rutaceae	
	olive	*Olea europaea* L.	Oleaceae	
Ornamental plants	carnation	*Dianthus* spp.	Caryophyllaceae	[10,11]
	bay laurel	*Laurus nobilis* L.	Lauraceae	
	cypress	*Cupressus* spp.	Cupressaceae	
	broom	*Cytisus* spp.	Fabaceae	
	daphne	*Daphne* spp.	Thymeleaceae	
	false acacia	*Robinia pseudoacacia* L.	Fabaceae	
	fuchsia	*Fuchsia* spp.	Onagraceae	
	spider flower	*Grevillea* spp.	Proteaceae	
	honeysuckle	*Lonicera* spp.	Caprifoliaceae	
	St. John’s wort	*Hypericum* spp.	Hypericaceae	
	ivy	*Hedera* spp.	Araliaceae	
	Japanese spindle	*Euonymus japonicus* Thunb.	Celastraceae	
	privet	*Ligustrum vulgare* L.	Oleaceae	
	laurustinus	*Viburnum tinus* L.	Adoxaceae	
	Japanese laurel	*Aucuba japonica* Thunb.	Garryaceae	
	Cape sundew	*Drosera capensis* L.	Droseraceae	[12]

## Data Availability

All data are included in the manuscript.
